# The prognostic value of tumor-associated macrophages detected by immunostaining in diffuse large B cell lymphoma: A meta-analysis

**DOI:** 10.3389/fonc.2022.1094400

**Published:** 2023-01-20

**Authors:** Mei Lin, Shupei Ma, Lingling Sun, Zhiqiang Qin

**Affiliations:** ^1^ Department of Pathology, School of Basic Medicine, Qingdao University, Qingdao, Shandong, China; ^2^ Department of Hematology, Qingdao Municipal Hospital, Qingdao, Shandong, China; ^3^ Department of Pathology, The Affiliated Hospital of Qingdao University, Qingdao, Shandong, China; ^4^ Department of Pathology, People Hospital of Changzhi, Changzhi, Shanxi, China

**Keywords:** diffuse large B cell lymphoma (DLBCL), prognosis, meta-analysis, tumor-associated macrophages (TAMs), M2 TAMs

## Abstract

**Background:**

The prognostic implication of tumor-associated macrophages (TAMs) in the microenvironment of diffuse large B cell lymphoma (DLBCL) remains controversial.

**Methods:**

A systematic and comprehensive search of relevant studies was performed in PubMed, Embase and Web of Science databases. The quality of the included studies was estimated using Newcastle-Ottawa Scale (NOS).

**Results:**

Twenty-three studies containing a total of 2992 DLBCL patients were involved in this study. They were all high-quality studies scoring ≥ 6 points. High density of M2 TAMs in tumor microenvironment significantly associated with both advanced disease stage (OR= 1.937, 95% CI: 1.256-2.988, P = 0.003) and unfavorable overall survival (OS) (HR = 1.750, 95% CI: 1.188-2.579, P = 0.005) but not associated with poor progression free survival (PFS) (HR = 1.672, 95% CI: 0.864-3.237, P = 0.127) and international prognostic index (IPI) (OR= 1.705, 95% CI: 0.843-3.449, P = 0.138) in DLBCL patients. No significant correlation was observed between the density of CD68^+^ TAMs and disease stage (OR= 1.433, 95% CI: 0.656-3.130, P = 0.366), IPI (OR= 1.391, 95% CI: 0.573-3.379, P = 0.466), OS (HR=0.929, 95% CI: 0.607-1.422, P = 0.734) or PFS (HR= 0.756, 95% CI: 0.415-1.379, P = 0.362) in DLBCL patients.

**Conclusion:**

This meta-analysis demonstrated that high density of M2 TAMs in the tumor microenvironment was a robust predictor of adverse outcome for DLBCL patients.

**Systematic review registration:**

https://www.crd.york.ac.uk/PROSPERO, identifier CRD42022343045.

## Background

Diffuse large B-cell lymphoma (DLBCL), the most common subtype of non-Hodgkin lymphoma (NHL), occupying 30-40% of newly diagnosed NHL (1, 2). DLBCL was heterogenous and patients with DLBCL showed various clinical outcomes (3). Approximately 60-70% DLBCL patients can be cured by anti-CD20 based immunochemotherapy. However, relapsed and refractory patients still die from DLBCL and its complications (4–6). Further improvement of DLBCL patients’ therapeutic outcome relies on identifying high-risk patients and individualizing treatment regimens.

Recent studies by molecular profiling showed that tumor microenvironment (TME) was associated with clinical behavior of DLBCL. Lenz and colleagues demonstrated that the prognosis of DLBCL patients was influenced by differences of TME. They also demonstrated that high stromal-2 signature predicted poor outcome (7). Using gene expression and sequencing, several other studies also obtained promising results in identifying high-risk DLBCL patients (8–12). However, molecular profiling has the disadvantage of low applicability in daily practice.

Tumor-associated macrophages (TAMs) are the most abundant component of TME (13). Recent studies have demonstrated that TAMs were critical for the survival, growth, metastasis, and drug resistance of tumors (14, 15). In response to different environmental stimuli, TAMs differentiated into M1 type (classically activated phenotype) and M2 type (alternatively activated phenotype) (16). The two types of TAMs were distinguished in functions and surface markers. M1 TAMs prevented tumor growth (17, 18). whereas M2 TAMs promoted angiogenesis and was involved in the progression of tumor (17–19). CD68 is a general marker for all TAMs and CD163 is a specific marker for M2 TAMs (3).

In DLBCL, the role of TAMs in the progression of DLBCL and the prognostic value of TAMs remains inconclusive due to the contradictory results obtained by previous studies. Several studies showed that a high density of CD68^+^ TAMs was associated with favorable prognosis (3, 20, 21). A few other studies failed to demonstrate such association (4, 18, 22–27). By contrast, Cai et al. (28) and Carreras et al. (17) showed that high density of CD68^+^ TAMs correlated with inferior outcome. The correlation between M2 TAMs and survival of DLBCL patients was also unsettled. Some researchers showed that a high density of M2 TAMs was correlated with shortened survival in DLBCL (1, 3, 17, 23). However, several other studies did not demonstrate such association (20, 24, 25, 27, 29–32). Therefore, we performed this meta-analysis to explore the role of TAMs in DLBCL progression and the prognostic value of TAMs in DLBCL patients.

## Methods

### Literature search

Relevant articles were systemically searched in PubMed, Embase and Web of Science databases with an end date of August 5th, 2022. The searching terms were “macrophage” or “macrophages” or “TAM” or “TAMs” or “tumor-infiltrating macrophage” or “tumor-associated macrophage” or “intratumoral macrophage” and “diffuse large B-cell lymphoma” or “DLBCL”. In addition, we also searched the references of relevant studies for eligibility. The literature search was performed by two independent reviewers (Mei Lin and Shupei Ma) and disagreement was resolved by consensus.

### Inclusion criteria

Our inclusion criteria were as follows (1): proven diagnosis of DLBCL; (2) CD68^+^ TAMs, CD163^+^ TAMs or CD163^+^/CD68^+^ TAMs were detected by immunohistochemical or immunofluorescence staining; (3) patients were categorized into high and low density TAMs groups; (4) Odds ratio (OR) or hazard ratio (HR) and 95% confidence interval (CI) on the density of CD68^+^, CD163^+^ or CD163^+^/CD68^+^ TAMs and disease stage, international prognostic index (IPI), overall survival (OS) or progression free survival (PFS) could be obtained.

### Data extraction and quality assessment

The data extraction was performed by two reviewers (Shupei Ma and Mei Lin) independently. For each eligible study, we extracted the following data: surname of the first author, year of publication, number of patients, country, treatment, median/mean/average follow-up, method, antibody (clone), analysis. For studies that HR and its 95% CI were not reported, the data was extracted using the Tierney’s calculation method (33). The Newcastle-Ottawa Scale (NOS) was used to assess the quality of the involved studies and any study scores ≥ 6 was considered as a high-quality literature.

### Statistical analysis

Pooled OR and 95% CI were used to estimate the correlation between the density of TAMs and disease stage or IPI. Pooled HR and 95% CI were used to investigate the effect of TAMs on prognosis. To evaluate the interstudy heterogeneity, chi-squared test (Q test) and I² test were used. P > 0.10 and I² < 50% indicated no significant heterogeneity existed. In this case, fixed-effect model was used. Otherwise, random-effect model was applied. Sensitivity analysis and subgroup analysis were applied to explore the source of heterogeneity. Publication bias was evaluated by funnel plot and Egger test. All statistical analysis was performed using Stata 12.0 software. P < 0.05 was considered statistically significant.

## Results

### Identification of eligible studies

**Figure 1:**
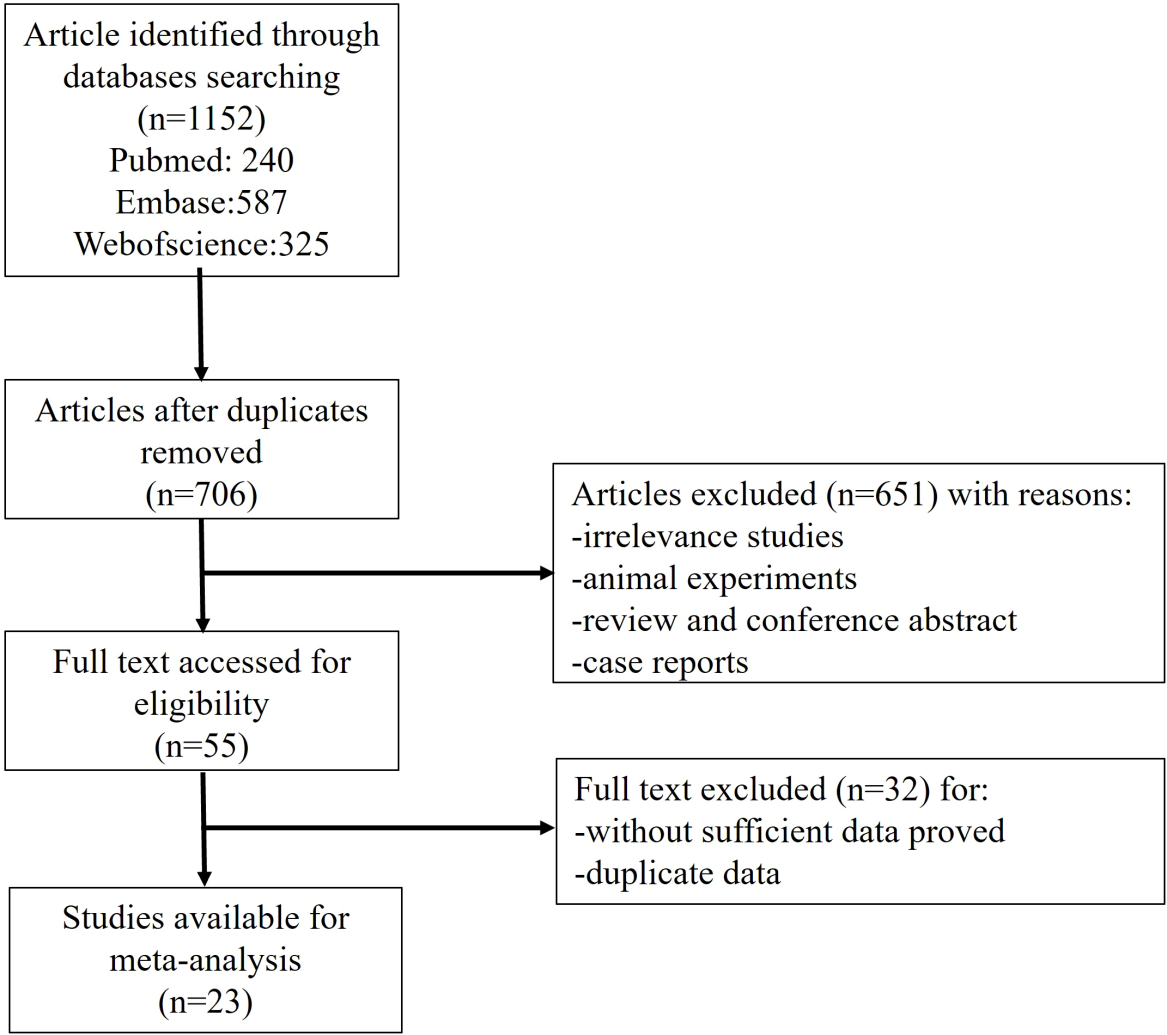
Flow chart of study selection.

A total of 1152 literatures were retrieved according to the abovementioned searching strategy, including 240 from PubMed, 587 from Embase and 325 from Web of Science. A total of 446 duplication was excluded. By carefully reviewing the title and abstract, we excluded 651 articles which are non-original, irrelevant or laboratory studies on animals or cell lines. The remaining 55 studies were further investigated by reading the full text carefully. Thirty-two studies were then excluded due to not fulfilling the inclusion criteria. Finally, 23 studies were eligible for this meta-analysis (**Figure 1**).

### Characteristics of included studies and quality assessment

The basic characteristics of the 23 eligible studies was shown in **Table 1**. The included studies were published between 2011-2022 and the number of participants ranged from 36 to 430. Of the 23 included studies, 5 were from Japan (17, 26, 27, 31, 32) and China (1, 23, 28, 34, 35) respectively; 4 from Korea (3, 20, 29, 36), 3 from USA (4, 22, 30) and Italy (21, 24, 37) respectively; 1 from Finland (18), India (25) and Egypt (38) respectively. Immunohistochemistry or immunofluorescence was performed by the included studies. Antibodies against CD68 was used to detect total TAMs and anti-CD163 antibody or double staining with antibodies against CD68 and CD163 was applied to estimate M2 TAMs by the eligible studies.

**Table 1:** Characteristics of the eligible studies.

Author	Year	N	Country	Treatment	Follow-up median/mean/average (months)	Method	Antibody (clone)	Analysis
Asano	2022	82	Japan	RT/RT+HD-MTX/R-MPV+RT/HD-MTX/R-MPV/others	ND	IHC	CD163(MRQ26)	OS, PFS
Cai	2012	112	China	CHOP/CHOP+RT	72 (2–135)	IHC	CD68(KP1)	OS, PFS
Carreras	2022	132	Japan	R-CHOP/R-CHOP- like/others	ND	IHC	CD68(514H12),CD163(10D6)	OS, PFS
Cencini	2020	37	Italy	CHOP-like/R-CHOP	60	IHC	CD68(PG-M1), CD163(ND)	OS,PFS,IPI,stage
Croci	2021	430	Italy	R-CHOP like	ND	IHC	CD68(PG-M1)	OS, PFS
Gomez-Gelvez	2016	70	USA	R-CHOP	49.2(7.2-144)	IHC	CD68(KP1)	OS, PFS
Jeong	2017	185	Korea	ND	38.7(mean)	IHC	CD163(MRQ26)	OS
Li	2019	221	China	CHOP/R-CHOP	42(3-118)	IHC	CD68(KP1), CD163(10D6)	OS,PFS,IPI,stage
Marchesi	2015	61	Italy	R-chop/R-chop like	24.7	IFA	CD163(ND)/CD68(ND)	OS
Matsuki	2019	94	USA	CT/R+CT	64.8	IHC	CD163(10D6)	OS, PFS
Meyer	2011	242	USA	R-CHOP/CHOP like	ND	IHC	CD68(KP1)	OS
Nam	2014	109	Korea	R-CHOP	43(16-178)	IHC	CD68(PG-M1), CD163(10D6)	OS, PFS
Nam	2018	144	Korea	MVP/HD-MTX/RT/R-MVP/others	31.35*(0.2-178)	IHC	CD68(PG-M1), CD163(10D6)	OS, PFS
Parkhi	2021	44	India	CT ± R/RT+CT ± R/RT/not received	ND	IHC	CD68(PG-M1),CD163(MRQ26)	OS
Riihijarvi	2015	181	Finland	CT/R+CT	65,65,85	IHC	CD68(KP1)	OS, PFS, IPI
Wada	2012	101	Japan	R+CT (most)	28^+^(9.5-38.5)	IHC	CD68 (PG-M1) CD163(ND)/CD68(PG-M1)	OS, IPI, stage
Wang	2017	355	China	R-CHOP	53.71	IHC	CD163(ND)	OS, PFS
Xu	2013	92	China	ND	ND	IHC	CD163(10D6)	OS, IPI
Yamamoto	2014	36	Japan	R-CHOP	37.2	IHC	CD163(10D6)	PFS
Yoshida	2013	47	Japan	R-chop/R+THP-COP	ND	IHC	CD68(KP1), CD163(10D6)	OS
Ghorab	2022	65	Egypt	ND	ND	IHC	CD68(KP1)	IPI
Wang	2015	81	China	R-CHOP	ND	IHC	CD68(514H12)	IPI, stage
Lee	2011	71	Korea	CT+R/CT/CT+RT/operation	45.6(6-132)	IHC	CD68(KP1)	IPI, stage

N: number of patients; RT, radiotherapy; HD, high-dose; MTX, methotrexate; R, rituximab; MPVA, MTX, procarbazine, vincristine, and Ara-C; MPV, MTX, procarbazine, vincristine; ND, not described; IHC, immunohistochemistry; IFA, immunofluorescence assay; OS, overall survival; PFS, progression free survival; MVP, methotrexate, vincristine, procarbazine; CHOP, cyclophosphamide, doxorubicin, vincristine, prednisone; CT, chemotherapy; THP-COP, pirarubicin, cyclophosphamide, vincristine, prednisolone; * median follow-up for the group of patients who did not receive rituximab; + average follow-up.

The quality of the 23 included studies was estimated by NOS. The scores were all ≥ 6 points (**Supplementary Table 1**). This suggested that all the eligible studies were high-quality studies.

### Total TAMs and IPI, disease stage or prognosis

**Figure 2:**
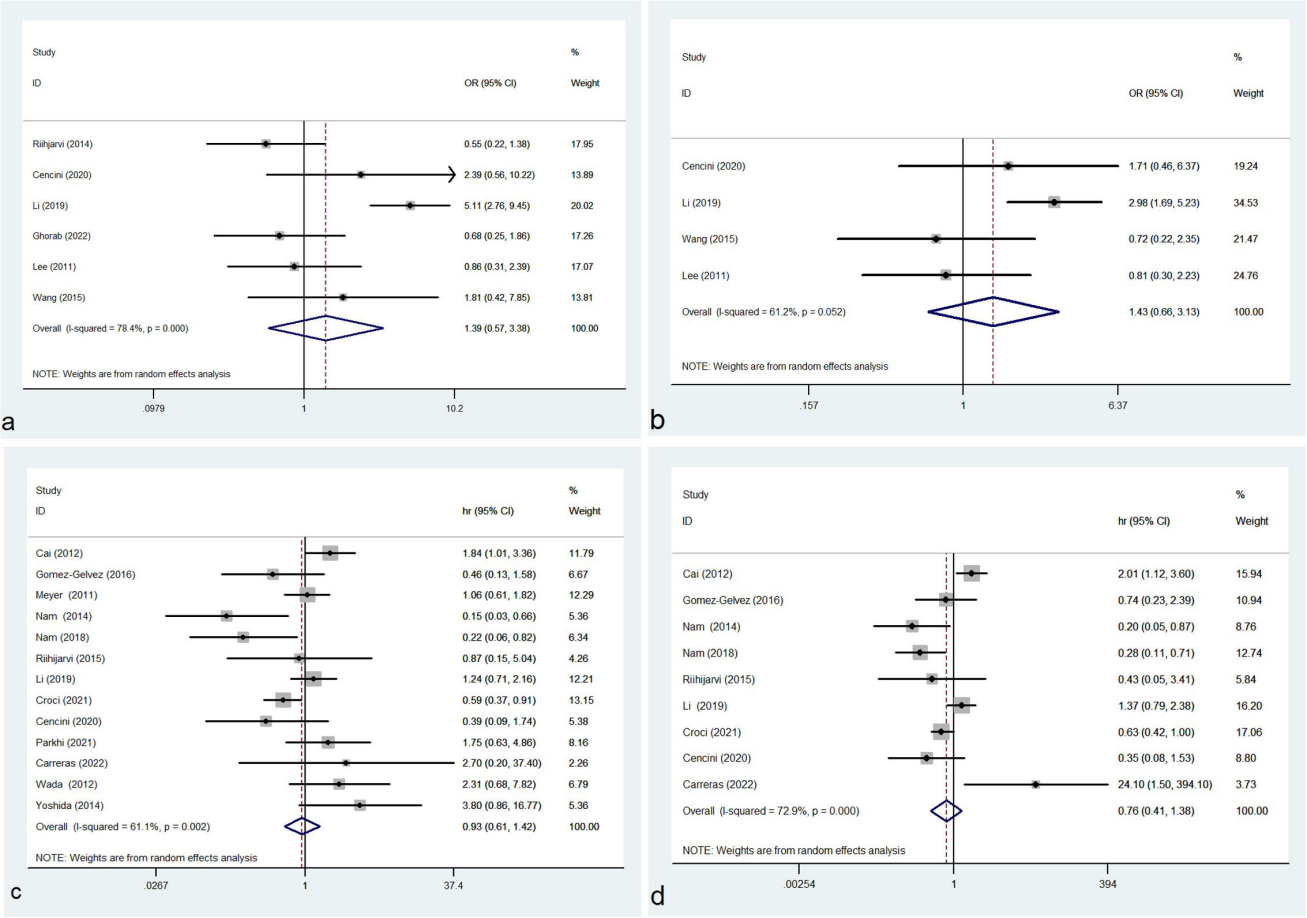
Forest plot of total TAMs and IPI **(A)**, disease stage **(B)**, OS **(C)** and PFS **(D)**; OR, odds ratio; hr, hazard ratio; CI, confidence interval.

In this study, the density of total TAMs was not correlated with IPI (≥3/0-2: OR= 1.391, 95% CI: 0.573-3.379, P= 0.466) with significant heterogeneity (P= 0.000, I² =78.4%) (**Figure 2A**). No correlation was observed between the density of total TAMs and disease stage (Ann Arbor stage, III+IV/I+II: OR= 1.433, 95% CI: 0.656-3.130, P = 0.366) with evident heterogeneity (P = 0.052, I² = 61.2%) (**Figure 2B**).

Of the 23 eligible studies, 13 or 9 studies reported the association between the density of CD68^+^ TAMs and OS or PFS respectively. In our meta-analysis, no significant correlation was observed between the density of total TAMs and OS (HR=0.929, 95% CI: 0.607-1.422, P = 0.734), with significant heterogeneity (P = 0.002, I² = 61.1%) (**Figure 2C**). No significant association was identified between the density of total TAMs and PFS (HR= 0.756, 95% CI: 0.415-1.379, P = 0.362) and the heterogeneity was significant (P = 0.000, I² = 72.9%) (**Figure 2D**).

### M2 TAMs and IPI, disease stage or prognosis

**Figure 3:**
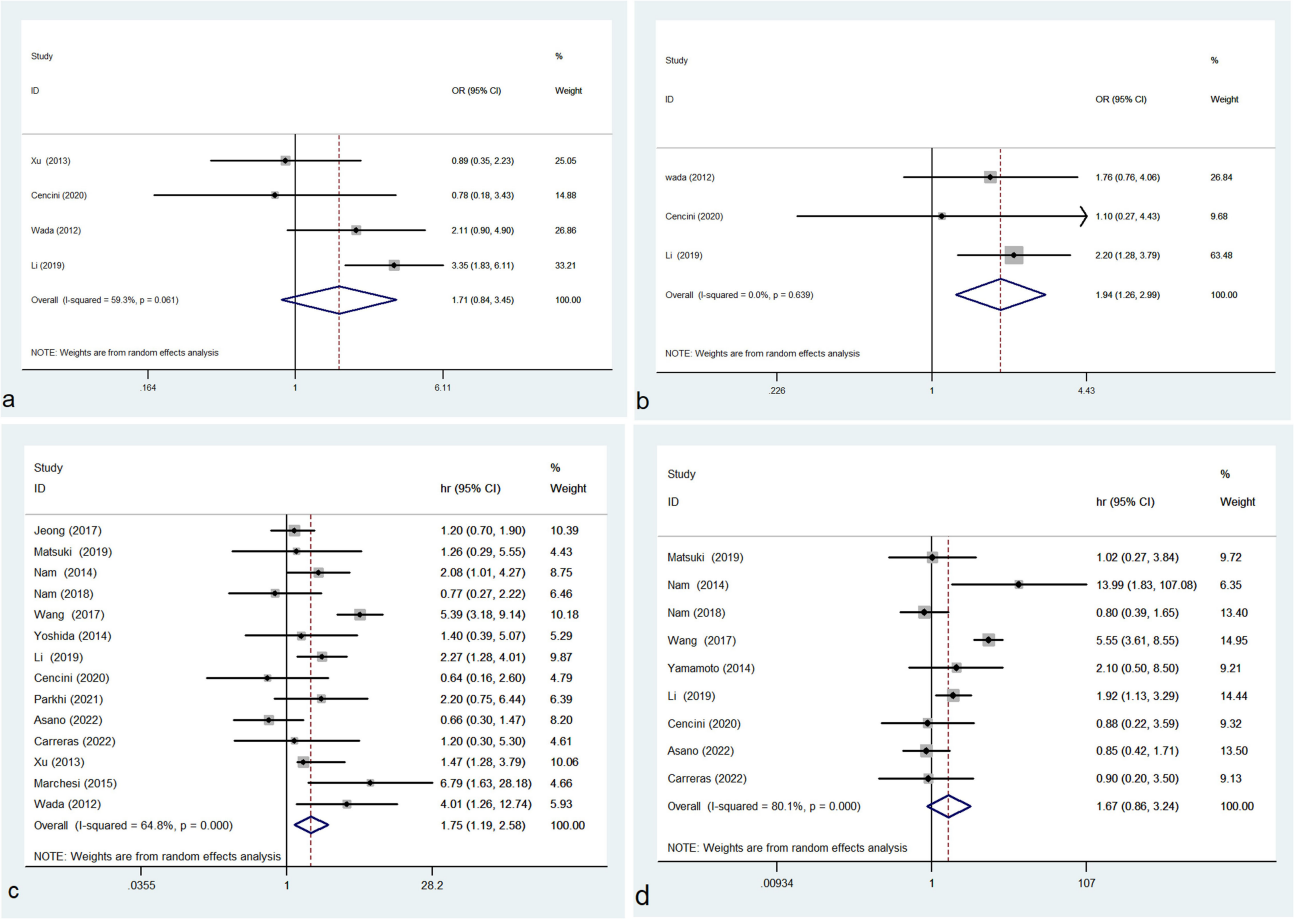
Forest plot of M2 TAMs and IPI **(A)**, disease stage **(B)**, OS **(C)** and PFS **(D)**; OR, odds ratio; hr, hazard ratio; CI, confidence interval.

In this study, no correlation was observed between the density of M2 TAMs and IPI (OR= 1.705, 95% CI: 0.843-3.449, P = 0.138) with evident heterogeneity (P = 0.061, I² = 59.3%) (**Figure 3A**). High density of M2 TAMs associated with disease stage (OR= 1.937, 95% CI: 1.256-2.988, P = 0.003) with no heterogeneity (P = 0.639, I² = 0.0%) (**Figure 3B**).

In this study, the pooled results of 14 studies showed that high density of M2 TAMs in the microenvironment of DLBCL patients correlated with unfavorable OS (HR = 1.750, 95% CI: 1.188-2.579, P = 0.005), with significant heterogeneity (P = 0.000, I² = 64.8%) (**Figure 3C**). Pooled HR for PFS in 9 studies showed that high density of M2 TAMs was not significantly associated with poor PFS (HR = 1.672, 95% CI: 0.864-3.237, P = 0.127), with evident heterogeneity (P = 0.000, I² = 80.1%) (**Figure 3D**).

### Sensitivity analysis

Sensitivity analysis was conducted by removing one study each time and recalculating the remaining studies (39). In the analysis of M2 TAMs and OS or PFS, the heterogeneity become insignificant (M2 TAMs and OS: P = 0.108, I² = 34.3%; M2 TAMs and PFS: P = 0.115, I²= 39.6%) after removing Wang et al.’s study (1). In the study of total TAMs and IPI or disease stage, there was no heterogeneity after removing Li et al.’s study (23). In the analysis of M2 TAMs and IPI, the heterogeneity become insignificant after removing Li et al.’s study (P = 0.306, I²= 15.7%) (23) or Xu et al.’s study (P = 0.179, I²= 41.9%) (34).

After removing Xu et al.’s study (34), high density of M2 TAMs was related to high and high-intermediate IPI (OR= 2.239, 95% CI: 1.140-4.396, P = 0.019). After removing Li et al.’s study (23), the density of M2 TAMs was not correlated with disease stage (OR=1.552, 95% CI: 0.758-3.180, P = 0.229). Except the abovementioned 2 studies, no other study significantly influenced the pooled results in this meta-analysis.

### Subgroup analysis

Subgroup analysis was performed in the studies of total TAMs or M2 TAMs and survival of DLBCL patients. The eligible studies were divided into two subgroups according to whether the study focused on central nervous system DLBCL (CNS DLBCL). Our results showed that high density of total TAMs was not correlated with OS in both CNS (HR=0.652, 95% CI: 0.087-4.881, P = 0.677) and non-CNS DLBCL patients (HR=0.974, 95% CI: 0.626-1.515, P = 0.906) (**Table 2**). A high density of total TAMs was associated with favorable PFS in CNS DLBCL patients (HR=0.275, 95% CI: 0.106-0.714, P = 0.008) but not in non-CNS patients (HR=0.881, 95% CI: 0.478-1.624, P = 0.684) (**Table 2**). High density of M2 TAMs correlated with poor OS (HR=2.038, 95% CI: 1.345-3.087, P = 0.001) and PFS (HR=2.195, 95% CI: 1.090-4.420, P = 0.028) in non-CNS DLBCL patients. The density of M2 TAMs was not correlated with both OS and PFS in CNS DLBCL patients (**Table 3**).

**Table 2:** Subgroup analysis of total TAMs and survival.

survival	Subgroups	Number of studies	Pooled results (95%CI)	P-value	Heterogeneity	
					P	I²
**OS**	**Site**					
	CNS DLBCL	2	0.652 (0.087-4.881)	0.677	0.015	83.2%
	Non-CNS DLBCL	11	0.974 (0.626-1.515)	0.906	0.006	59.5%
	**Region**					
	Asian	8	1.162 (0.615-2.198)	0.643	0.006	65.0%
	Non-Asian	5	0.704 (0.511-0.971)	0.032	0.421	0.0%
	**Treatment**					
	With Rituximab	6	0.546(0.256-1.164)	0.117	0.072	50.5%
	Without Rituximab	3	1.194 (0.404-3.523)	0.748	0.023	73.5%
	**Clone of antibody**					
	KP1	6	1.269 (0.866-1.862)	0.222	0.239	26.1%
	PG-M1	6	0.609 (0.289-1.283)	0.192	0.012	66.1%
**PFS**	**Site**					
	CNS DLBCL	1	0.275 (0.106-0.714)	0.008	–	–
	Non-CNS DLBCL	8	0.881 (0.478-1.624)	0.684	0.001	70.2%
	**Region**					
	Asian	5	0.972 (0.360-2.625)	0.955	0.000	82.2%
	Non-Asian	4	0.604 (0.410-0.890)	0.011	0.858	0.0%
	**Treatment**					
	With Rituximab	5	0.410 (0.219-0.769)	0.005	0.145	41.4%
	Without Rituximab	3	1.145 (0.336-3.904)	0.828	0.002	84.5%
	**Clone of antibody**					
	KP1	4	1.391(0.881-2.195)	0.157	0.281	21.6%
	PG-M1	4	0.421(0.244-0.729)	0.002	0.239	28.8%

OS, overall survival; PFS, progression free survival; CI, confidence interval; CNS-DLBCL, primary diffuse large B cell lymphoma of the central nervous system

**Table 3:** Subgroup analysis of M2 TAMs and survival.

survival	Subgroups	Number of studies	Pooled results (95%CI)	P-value	Heterogeneity	
					P	I²
**OS**	**Site**					
	CNS DLBCL	3	0.982 (0.480-2.008)	0.961	0.194	39.0%
	Non-CNS DLBCL	11	2.038 (1.345- 3.087)	0.001	0.003	62.7%
	**Region**					
	Asian	11	1.751 (1.158-2.646)	0.008	0.001	68.0%
	Non-Asian	3	1.760 (0.437- 7.083)	0.426	0.059	64.7%
	**Treatment**					
	With R	5	2.620 (1.232-5.572)	0.012	0.021	65.5%
	Without R	2	1.197 (0.363-3.952)	0.768	0.221	33.3%
	**Antibody**					
	CD163	12	1.549 (1.038-2.313)	0.032	0.001	65.3%
	CD163+CD68 double staining	2	4.941 (2.012-12.129)	0.000	0.573	0.0%
**PFS**	**Site**					
	CNS DLBCL	2	0.822 (0.496-1.362)	0.447	0.912	0.0%
	Non-CNS DLBCL	7	2.195 (1.090- 4.420)	0.028	0.002	71.6%
	**Region**					
	Asian	7	1.915 (0.897-4.089)	0.093	0.000	83.8%
	Non-Asian	2	0.951 (0.364 – 2.487)	0.919	0.881	0.0%
	**Treatment**					
	With R	4	3.475 (1.210-9.985)	0.021	0.068	58.0%
	Without R	2	0.891 (0.459-1.731)	0.734	0.453	0.0%

OS, overall survival; PFS, progression free survival; CI, confidence interval; CNS DLBCL: primary diffuse large B cell lymphoma of the central nervous system; R, rituximab.

Based on geographic region, the included patients were classified into Asian group and non-Asian group. High density of total TAMs correlated with favorable OS (HR=0.704, 95% CI: 0.511-0.971, P = 0.032) and PFS (HR=0.604, 95% CI: 0.410-0.890, P = 0.011) in non-Asian patients with no heterogeneity (total TAMs and OS: P = 0.421, I² = 0.00%; total TAMs and PFS: P = 0.858, I² = 0.00%). However, the density of total TAMs was not correlated with both OS and PFS in Asian patients (**Table 2**). High density of M2 TAMs associated with poor OS (HR=1.751, 95% CI: 1.158-2.646, P = 0.008) and showed a trend of association with poor PFS (HR=1.915, 95% CI: 0.897-4.089, P = 0.093) in Asian patients. The density of M2 TAMs was not correlated with both OS and PFS in non-Asian patients (**Table 3**).

In the subgroup analysis according to whether rituximab was included in the treatment regimen, a high density of total TAMs was significantly correlated with favorable PFS (HR=0.410, 95%CI: 0.219-0.769, P=0.005) but not significantly correlated with OS (HR=0.546, 95% CI: 0.256-1.164, P = 0.117) in patients treated with rituximab-containing regimen (**Table 2**). In contrast, no correlation was observed between the density of total TAMs and OS or PFS in patients treated without rituximab (**Table 2**). High density of M2 TAMs significantly correlated with unfavorable OS (HR=2.620, 95% CI: 1.232-5.572, P = 0.012) and PFS (HR=3.475, 95% CI: 1.210-9.985, P = 0.021) in patients treated with rituximab-containing regimen. However, no correlation was found between the density of M2 TAMs and prognosis of patients treated without rituximab (**Table 3**).

Subgroup analysis according to different clones of anti-CD68 antibody was also performed. In the subgroup detected CD68 with clone PG-M1, total TAMs correlated with PFS (HR=0.421, 95% CI: 0.244-0.729, P = 0.002) but not correlated with OS (HR=0.609, 95% CI: 0.289-1.283, P = 0.192) (**Table 2**). In KP1 subgroup, no correlation was observed between total TAMs and OS (HR=1.269, 95% CI: 0.866-1.862, P = 0.222) or PFS (HR=1.391, 95% CI: 0.881-2.195, P = 0.157) (**Table 2**).

Among the 14 studies reported the correlation between M2 TAMs and OS, 12 studies used anti-CD163 antibody and 2 studies applied double staining with antibodies against CD163 and CD68 to estimate M2 TAMs. The density of CD163^+^ TAMs and CD163^+^/CD68^+^ TAMs was both correlated with OS (CD163^+^ TAMs: HR=1.549, 95% CI: 1.038-2.313, P = 0.032; CD163^+^/CD68^+^ TAMs: HR=4.941, 95% CI: 2.012-12.129, P = 0.000) (**Table 3**).

### Publication bias

Funnel plots and Egger tests were used to assess the publication bias. The funnel plots of this meta-analysis were shown in **Supplementary Figure 1** and **Supplementary Figure 2**. The yielded P values of Egger test were: 0.180 for total TAMs and disease stage, 0.302 for total TAMs and IPI, 0.860 for total TAMs and OS, 0.707 for total TAMs and PFS, 0.018 for M2 TAMs and disease stage, 0.150 for M2 TAMs and IPI, 0.598 for M2 TAMs and OS, 0.321for M2 TAMs and PFS. The results of Egger test showed that there was publication bias in the analysis of M2 TAM and disease stage. Except this, no publication bias existed in the other analysis of this meta-analysis.

## Discussion

Previous studies showed that TME is critical for the progression of tumors (14). TAMs are important component of TME (28). The prognostic significance of total TAMs and M2 TAMs have been investigated in a variety of cancers by meta-analysis (40–44). In lymphoma, meta-analysis investigated the association of total TAMs or M2 TAMs and outcome of patients have been reported in non-Hodgkin’s lymphoma (NHL) (45) and Hodgkin’s lymphoma (HL) (46). DLBCL is the most common type of NHL. However, meta-analysis investigating the prognostic value of TAMs in DLBCL is still unavailable.

Consistent with the results obtained by meta-analysis in HL (46), gastric cancer (40) and NHL (45), we demonstrated that high density of M2 TAMs correlated with unfavorable prognosis in DLBCL. This suggested that high density of M2 TAMs can be used as an indicator of poor prognosis in DLBCL patients. Xu et al.’s study reported that high density of CD68^+^ TAMs correlated with poor OS and PFS in NHL (45) and several previous studies suggested that TAMs’ infiltration was significantly correlated with favorable (3, 20, 21) or poor outcome (17, 28) in DLBCL. However, no correlation between the density of CD68^+^ TAMs and prognosis in DLBCL patients was found in this meta-analysis. This was in accordance with previous studies in bladder (41) and ovarian cancers (42). Taken together, these results suggested that M2 TAMs rather than total TAMs might contribute to the progression of DLBCL and lead to unfavorable outcome in DLBCL patients.

In this study, high density of M2 TAMs was correlated with unfavorable prognosis in Asian subgroup but not in non-Asian subgroup. These suggested that M2 TAMs may play an important role in the disease progression and acted as an indicator of poor prognosis in Asian patients. In this meta-analysis, high density of CD68^+^ TAMs associated with favorable outcome in non-Asian DLBCL patients but not in Asian patients. This suggested that high density of CD68^+^ TAMs predicted favorable survival in non-Asian patients but not in Asian patients.

Rituximab, a human/murine chimeric antibody, shows high affinity and specificity for CD20 which is a transmembrane protein of B-lymphocyte. Rituximab has become a standard component of treatment modality for a number of B-cell malignancies including DLBCL (47). Taskinen and colleagues reported that addition of rituximab to the same group of patients at relapse reversed the negative prognostic effect of high density CD68^+^ TAMs in tumor environment to favorite (48). In this meta-analysis, high density of CD68^+^ TAMs correlated with favorable outcome in the subgroup of patients treated with rituximab-containing regimen. In contrast, no correlation was found between high density of CD68^+^ TAMs and prognosis in patients treated without rituximab. These results were in accordance with previous study and suggested that TAMs might obtain tumor-inhibiting function in response to rituximab or TAMs modulated the therapeutic efficiency of rituximab.

The results of our meta-analysis showed that the association of M2 TAMs and outcome of DLBCL patients was also influenced by whether rituximab was included in the treatment regimen. Pooled results of this meta-analysis showed that high density of M2 TAMs in tumor microenvironment associated with unfavorable outcome in patients treated with rituximab. In contrast, no correlation was observed between the density of M2 TAMs and prognosis in patients treated without rituximab. This emphasized the importance of targeting M2 macrophage in rituximab era.

TAMs centered therapeutic strategies includes suppressing the recruitment of TAMs, depletion of TAMs and reprogramming M2 TAMs to M1 type (49). Administration of antibody against chemokine (C-C motif) ligand-2 (CCL2) led to decreased infiltration of TAMs and impacted tumor growth in animal models of human cancers (50, 51). Colony stimulating factor 1 (CSF-1) is a major factor for the survival of TAMs. Targeting CSF-1 receptor with a humanized antibody RG7155 led to obvious reduction of TAMs in various tumor tissues (52). Maeda et al. showed that toll-like receptor 3 (TLR3) agonist Poly (I:C) was effective in reprogramming macrophage to anti-tumor type by an *in vitro* study (53). Repolarization of TAMs can also be achieved through manipulation of CD40 (54) and CD47 pathways (55). Currently, a variety of antibodies against CD40 (56) or CD47 (57) are being evaluated in clinical trials. A phase IIb clinical trial was performed to investigate the therapeutic efficacy of anti-CD40 antibody dacetuzumab plus rituximab, ifosfamide, carboplatin, and etoposide in 151 patients with relapsed and refractory DLBCL. The complete remission (CR) rate of the dacetuzumab group was not superior compared to the group using placebo in place of dacetuzumab (58). A phase Ib/II clinical trials of anti-CD47 antibody Hu5F9-G4 combined with rituximab in 75 patients with relapsed and refractory lymphoma showed promising results (57). In DLBCL patients treated with rituximab-containing regimen, the pooled results of this meta-analysis showed that high density of total TAMs significantly correlated with favorable outcome while high density of M2 TAMs significantly associated with poor prognosis. This suggested that repolarization of TAMs from M2 to M1 might have more clinical benefit than the methods of merely reducing the number of M2 TAMs in the treatment of DLBCL patients who received rituximab-containing regimen.

In the subgroup analysis according to different clones of anti-CD68 antibody, high density of total TAMs correlated with favorable PFS in the subgroup using clone PG-M1. While no association was identified between total TAMs and OS or PFS in KP1 subgroup. These suggested that high density of total TAMs detected by PG-M1 rather than KP1 was an indicator of favorable prognosis in DLBCL patients.

The current study is the first systemic meta-analysis investigating the association between the density of total TAMs or M2 TAMs and prognosis in DLBCL patients. However, several limitations in this study need to be addressed. First, some of the involved studies did not report HR. We extracted data from the Kaplan-Meier curves of these studies. In this case, deviation from the real value of HR may be caused. Second, the treatment of DLBCL patients in the included studies are variable. This may influence the survival of patients and contribute to heterogeneity. Third, significant heterogeneity exists in this meta-analysis. The interstudy heterogeneity might be derived from the differences in origin of patients, sample size, location of locus, tumor stages, inconsistency of cut-off value and the antibody used to estimate TAMs. Forth, significant publication bias was observed in the study of M2 TAMs and disease stage in this meta-analysis. This may be due to that studies with positive results are more likely to be published than those reporting negative results. In addition, only three studies were eligible for this analysis. Therefore, more studies are needed to verify our results.

## Conclusion

This meta-analysis demonstrated that a high density of M2 TAMs was a robust predictor of unfavorable outcome for DLBCL patients.

## Data availability statement

The original contributions presented in the study are included in the article/**Supplementary Material**. Further inquiries can be directed to the corresponding author.

## Author contributions

ML designed the study and revised the manuscript, ML, SM, LS, and ZQ selected the study, extracted and analyzed the data and wrote the manuscript. All authors contributed to the article and approved the submitted version.

## Supplementary material

Supplementary Figure 1 Funnel plot for publication bias of total TAMs and IPI **(A)**, disease stage **(B)**, OS **(C)** and PFS **(D)**.

Supplementary Figure 2 Funnel plot for publication bias of M2 TAMs and IPI **(A)**, disease stage **(B)**, OS **(C)** and PFS **(D)**.

Supplementary Table 1 Newcastle-Ottawa Quality Assessment Scale. *** A study can be awarded a maximum of one star for each numbered item within the Selection and Outcome categories. A maximum of two stars can be given for Comparability. http://www.ohri.ca/programs/clinical_epidemiology/oxford.asp
