# Supplementary figures and images for: The prognostic value of tumor-associated macrophages detected by immunostaining in diffuse large B cell lymphoma: A meta-analysis
Source: Front Oncol. 2023 Jan 20;12:1094400. doi: 10.3389/fonc.2022.1094400 (PMC9895774; doi:10.3389/fonc.2022.1094400)

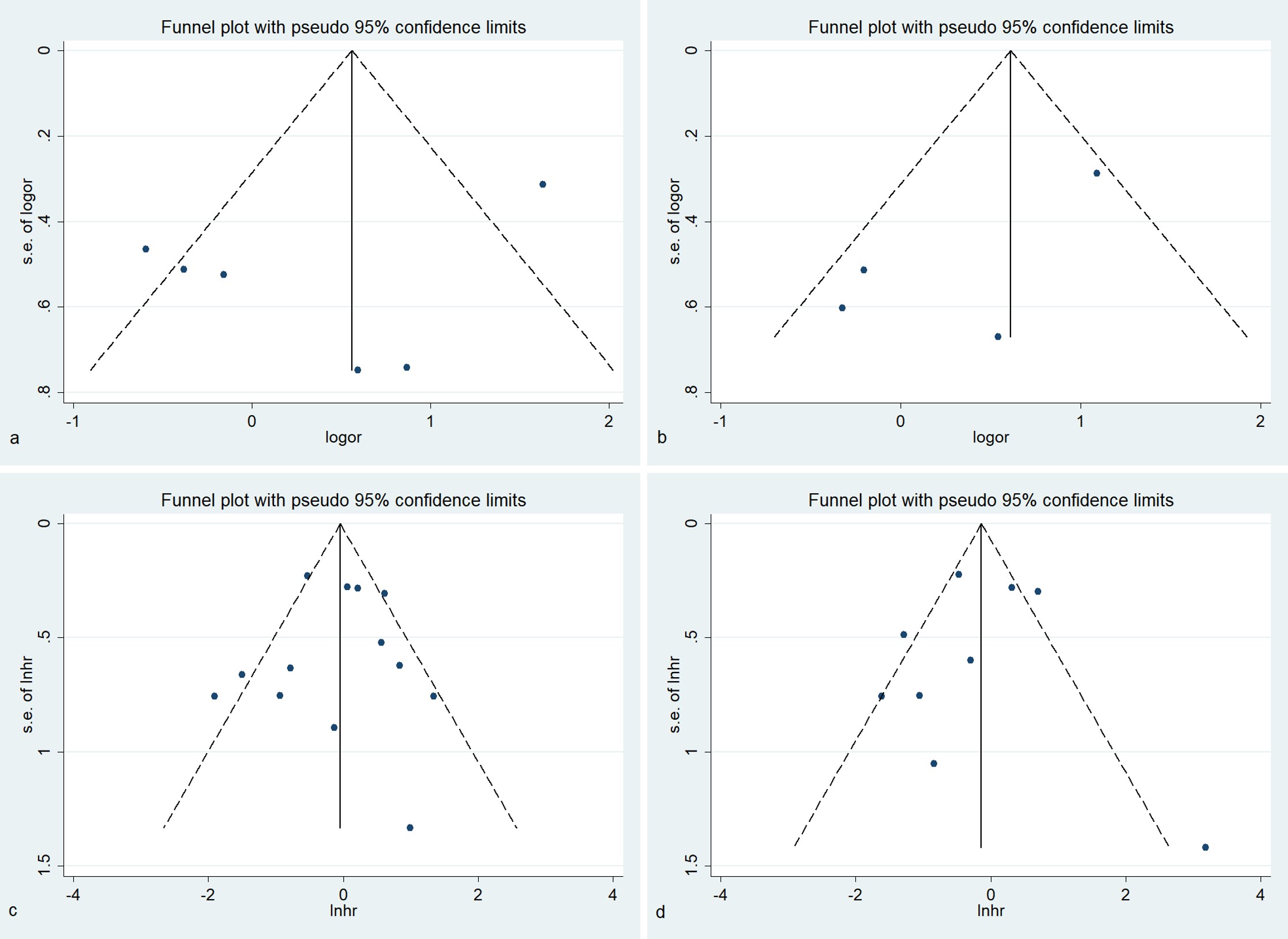

Supplement: Supplementary Figure 1 — Funnel plot for publication bias of total TAMs and IPI (A), disease stage (B), OS (C) and PFS (D). [file Image_1.jpg]

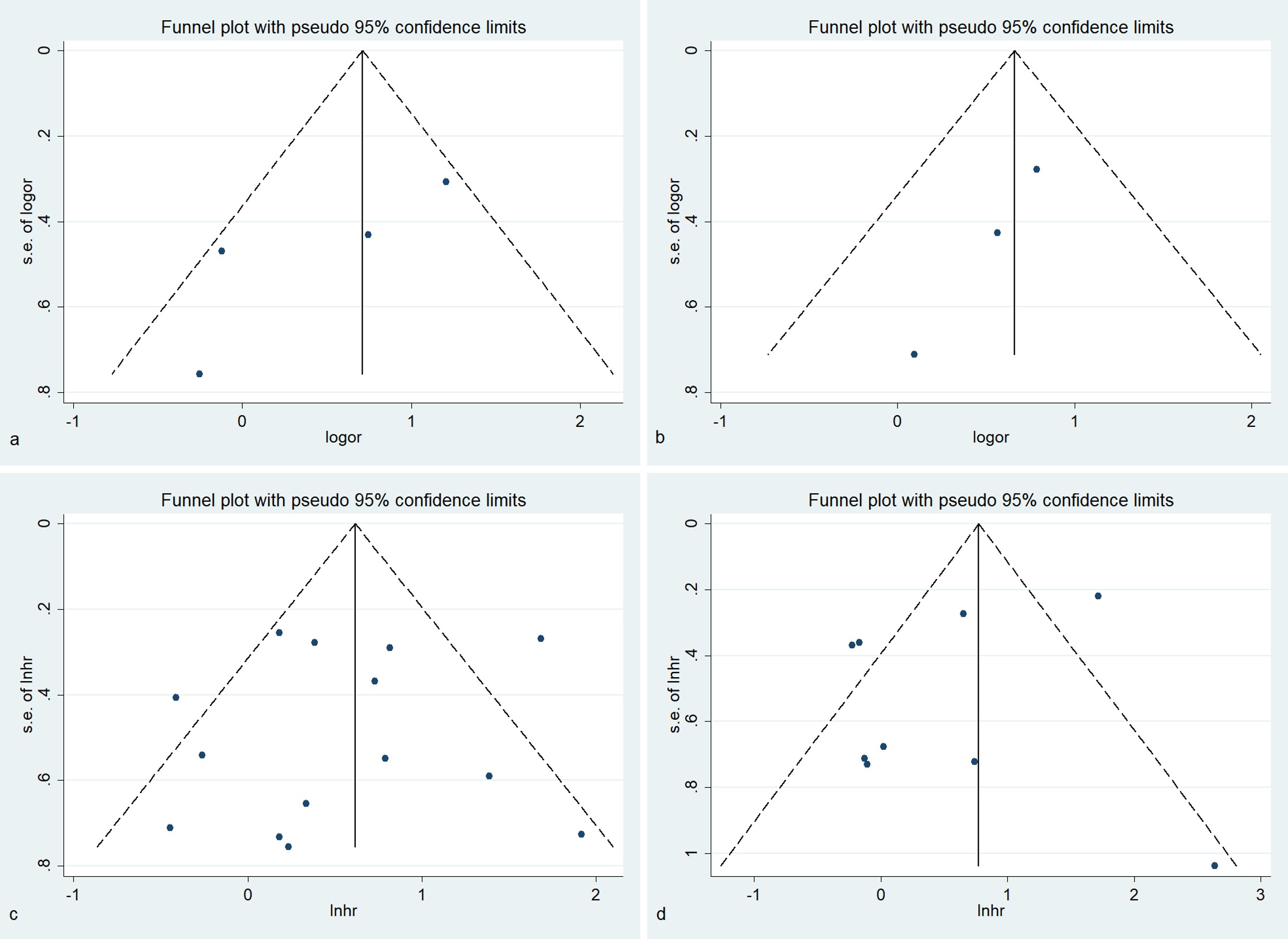

Supplement: Supplementary Figure 2 — Funnel plot for publication bias of M2 TAMs and IPI (A), disease stage (B), OS (C) and PFS (D). [file Image_2.jpg]
