# Supplementary material for: The prognostic value of tumor-associated macrophages detected by immunostaining in diffuse large B cell lymphoma: A meta-analysis
Source: Front Oncol. 2023 Jan 20;12:1094400. doi: 10.3389/fonc.2022.1094400 (PMC9895774; doi:10.3389/fonc.2022.1094400)
Supplement: Supplementary Table 1 — Newcastle-Ottawa Quality Assessment Scale. * A study can be awarded a maximum of one star for each numbered item within the Selection and Outcome categories. A maximum of two stars can be given for Comparability. http://www.ohri.ca/programs/clinical_epidemiology/oxford.asp [file Table_1.pdf]

Supplementary Table 1. Newcastle-Ottawa Quality Assessment Scale

Study	Selection				Comparability	Outcome			Total Score
	exposed cohort	non exposed cohort	Ascertainment of exposure	Outcome of interest	Control for factor	Assessment of outcome	Follow-up long enough	Adequacy of follow-up	
Asano		*	*	*	**	*	*		7
Cai	*	*	*	*	**	*	*		8
Carreras	*	*	*	*	**	*	*		8
Cencini	*	*	*	*	**	*	*		8
Croci		*	*	*	**	*	*		7
Gomez-Gelvez	*	*	*	*	**	*	*	*	9
Jeong	*	*	*	*		*	*		6
Li	*	*	*	*		*	*	*	7
Marchesi	*	*	*	*		*	*		6
Matsuki	*	*	*	*	**	*	*		8
Meyer	*	*	*	*	**	*	*		8
Nam (2014)	*	*	*	*	*	*	*		7
Nam (2018)	*	*	*	*	*	*	*		7
Parkhi	*	*	*	*	**	*	*		8
Riihijarvi		*	*	*	**	*	*		7
Wada	*	*	*	*		*	*		6
Wang (2017)	*	*	*	*	**	*	*	*	9
Xu	*	*	*	*	*	*	*	*	8
Yamamoto	*	*	*	*	**	*	*		8
Yoshida	*	*	*	*	**	*	*		8
Ghorab	*	*	*	*	**	*	*	*	9
Wang (2015)	*	*	*	*	*	*	*	*	8
Lee	*	*	*	*	**	*	*	*	9

* A study can be awarded a maximum of one star for each numbered item within the Selection and Outcome categories. A maximum of two stars can be given for

Comparability. http://www.ohri.ca/programs/clinical_epidemiology/oxford.asp
